# Paradoxical response in ocular bartonellosis

**DOI:** 10.1007/s12348-011-0046-6

**Published:** 2011-11-05

**Authors:** Eran Zimran, Smadar Shilo, Tatiana Florescu, Shlomo Dotan, Shay Balag, Dror Mevorach, Oren Shibolet, Radgonde Amer

**Affiliations:** 1 Department of Internal Medicine, Hadassah University Hospital, POB 12000, Jerusalem, 91120 Israel; 2 Department of Ophthalmology, Hadassah University Hospital, POB 12000, Jerusalem, 91120 Israel

## Introduction

Cat scratch disease is an infectious disease caused by the bacteria *Bartonella henselae* [1] and is transmitted by cat scratches or exposure to cat saliva. The spectrum of its ocular manifestations includes Parinaud's oculoglandular syndrome, iritis, vitritis, neuroretinitis, panuveitis, retinitis appearing as small white intraretinal infiltrates, and choroidal white lesions [1, 2]. *B. henselae* is reported to be the most common cause of neuroretinitis; typically characterized by optic disk edema in association with a partial or complete macular star. The majority of infected patients presenting with neuroretinitis reported in the literature are treated with antibiotics. In some cases steroids were added. However several reports showed that even patients who did not receive treatment had an excellent visual outcome [1]. We describe here a paradoxical response to treatment in a young female presenting with bilateral neuroretinitis and inflammatory optic disk lesions.

## Case report

**Fig. 1:**
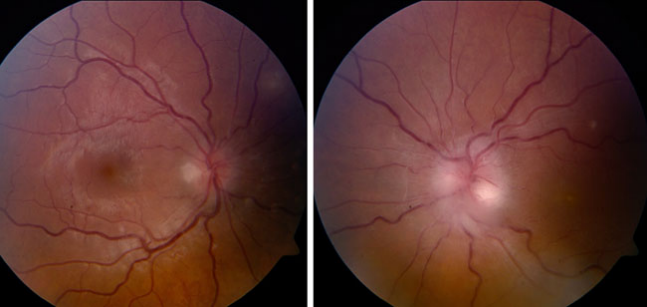
Color fundus photograph of the right eye showing a small white optic disk lesion in its temporal aspect with a deep retinal white lesion along the superonasal arcade (*left*). Color fundus photograph of the left eye showing blurred optic disk margins with white lesions in its nasal and temporal aspects with an associated localized vitreous opacity on its surface. Marked macular edema is also seen with a deep retinal white lesion along the superotemporal arcade (*right*)

**Fig. 2:**
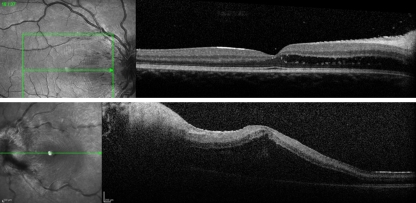
OCT showing intraretinal fluid in the right papillomacular bundle (*upper*) and left exudative macular detachment (*lower*)

**Fig. 3:**
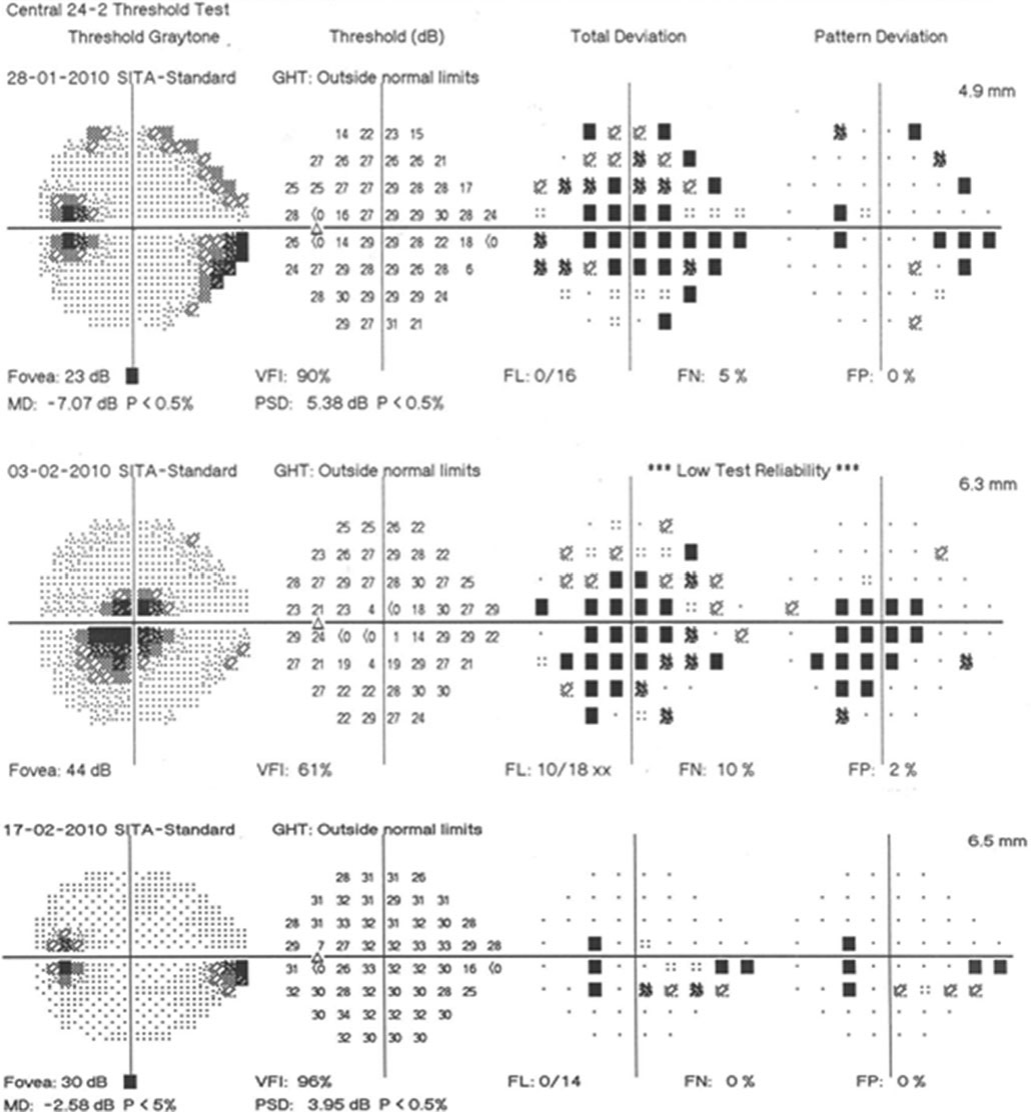
Visual field tests (24–2) showing enlargement of the left blind spot with nasal step on presentation (*upper*). Left central scotoma with inferotemporal extension was demonstrated few days later (*middle*). Total resolution of the central scotoma and improvement of the nasal step was noted 14 days later (*lower*)

A 19-year-old healthy female presented with an abrupt deterioration of left eye vision of 1-day duration. For 10 days prior to presentation, she was suffering from dry cough; 5 days later she had intermittent fever up to 41°C with shaking chills. There was a history of contact with a cat, but she denied being scratched. On admission, physical and neurologic examinations were unremarkable. Ophthalmologic assessment disclosed visual acuity of 4/4 in the right eye and 4/60 in the left eye. She had normal anterior segments and intraocular pressures. A left afferent pupillary defect was noted. Funduscopy of the right eye showed a small white optic disk lesion in its temporal aspect with a deep retinal white lesion along the superonasal arcade (Fig. 1). The left optic disk had blurred margins with bigger white lesions in its nasal and temporal aspects with an associated localized vitreous opacity on its surface (Fig. 1). There was marked macular edema and two deep retinal white lesions along the superotemporal arcade and inferonasal to the optic disk. Optical coherence tomography (OCT) revealed intraretinal fluid in the right papillomacular bundle and left exudative macular detachment (Fig. 2). Visual field examination showed right nasal step and superonasal arcuate defect while there was enlargement of the left blind spot with nasal step (Fig. 3). Work-up revealed mild anemia with hemoglobin of 11.7 g% and leukocytosis of 14,800 (lymphocytes, 31%). Kidney and liver functions were within normal limits except for a slightly elevated lactate dehydrogenase at 768 (normal range, 300–620 u/l). She had elevated erythrocyte sedimentation rate (ESR) (90 mm/h) and C-reactive protein (5.3 mg%; normal range, less than 1). Chest X-ray did not reveal any pathological findings, and CT scan and MRI of the head and brain showed mild thickening of the optic nerves bilaterally and hyperintense foci in the flair sequences in the intraocular aspects of the optic nerves, respectively. Cerebrospinal fluid cytology was normal. Serological tests were negative for HIV, syphilis, *Brucella*, *Coxiella burnetti*, toxoplasma, and toxocara. Serological tests for *B. henselae* revealed positive IgM titer at 75 units (negative, below 40) and IgG titer at 94 units (negative, below 60). Treatment was started with a combination of Doxycycline (100 mg twice daily) and Rifampicin (300 mg twice daily); in addition she received oral steroids at 1 mg/kg/day. Over the ensuing 3 days, she felt better, there was no fever, and visual acuity in the left eye improved to 4/24. Fundoscopy disclosed few hard exudates in the right papillomacular bundle and a left macular star while optic disk lesions were smaller bilaterally. OCT revealed marked resolution of bilateral macular edema. Four days following institution of therapy, she complained of a central defect in the left visual field. Visual acuity was counting fingers at 1 m in the left eye while it was unchanged in the right eye. Funduscopy did not reveal any new findings. Visual fields however revealed a left central scotoma (Fig. 3). Treatment with oral steroids was changed to intravenous Methylprednisolone (500 mg/day) for three consecutive days with a tapering regimen of oral steroids thereafter. There was prompt response with improvement in visual acuity and visual fields. Two weeks following the treatment, VA was 4/16 (left eye; LE). In addition, hemoglobin was 13.8 g% with a normal LDH at 366 u/l, WBC count was 11,900 (lymphocytes 15.4%), ESR was 28 mm/h, and CRP was less than 0.5. Treatment was discontinued after 10 weeks. After a 5-month follow-up period, vision was 4/4 in RE and 4/5 in LE. Visual fields were normal in RE but a small nasal step persisted in LE. Funduscopy was normal in the right eye but few small hard exudates were still seen in the left eye.

## Discussion

We herein describe the development of paradoxical response to treatment in a patient with serologically confirmed ocular bartonellosis. The patient developed central scotoma in one eye 4 days following the institution of anti-*Bartonella* and steroid therapy and following clinical improvement. We speculate that the initiation of the medical therapy led to a further compromise of the optic nerve function consequent to the overwhelming inflammatory reaction that sequentially settled following the use of high-dose steroid therapy.

Paradoxical response following initiation of therapy is also known as Jarisch–Herxheimer reaction (JHR) and both Jarisch and Herxheimer observed this reaction in patients suffering from syphilis who were being treated with mercury. It is now described in association with a variety of microbial infections, such as borreliosis, trichinellosis, Q fever, leptospiral infections, brucellosis, typhoid fever, and myalgic encephalomyelitis. It is classically described as being a systemic inflammatory response syndrome characterized by fever, chills, headache, myalgia, and exacerbation of skin lesions. No systemic involvement occurred in our patient; however, this reaction was limited to the deterioration in visual functions. Cheung and Chee reported on the occurrence of ocular disease (panuveitis and retinitis) with no systemic involvement following the institution of anti-tuberculous therapy for biopsy-proven tuberculous cervical lymphadenitis [3]. Prompt improvement was achieved with the addition of oral steroids. Fathilah and Choo described worsening of ocular disease following the institution of therapy for ocular syphilis [4]. To our knowledge, this is the first report of a paradoxical reaction occurring in a patient with ocular bartonellosis.

The pathophysiologic features of JHR are hypothesized to be a consequence of endotoxin reaction; however, recent studies showed that JHR is preceded by a cytokine surge with elevation of serum levels of tumor necrosis factor (TNFα), interleukin-6, and interleukin-8 [5]. Fekade et al. were successful in showing that JHR can be suppressed by anti-TNFα antibodies [6]. The favorable response to corticosteroids in patients with JHR [7] may result from transcriptional downregulation of TNFα, IFNɤ, and IL6 [8].

Our patient showed remarkable improvement in visual function following the institution of high-dose steroid therapy. We speculate that oral steroids that were initially instituted were insufficiently effective in obliterating the inflammatory cascade associated with the ocular infection, and only following the use of high-dose steroids was the paradoxical reaction reversed and persistent improvement consequently ensued.

## Conclusion

Paradoxical response has been described in association with a variety of microbial infections, such as borreliosis, trichinellosis, Q fever, leptospiral infections, brucellosis, and typhoid fever. To our knowledge, this is the first report of a paradoxical reaction occurring in a patient with ocular bartonellosis. High-dose steroids were needed in order to suppress the overwhelming inflammation associated with the response to treatment.
